# What Predicts Online Health Information-Seeking Behavior Among Egyptian Adults? A Cross-Sectional Study

**DOI:** 10.2196/jmir.6855

**Published:** 2017-06-22

**Authors:** Mayada Ghweeba, Antje Lindenmeyer, Sobhi Shishi, Mostafa Abbas, Amani Waheed, Shaymaa Amer

**Affiliations:** ^1^ Institute of Applied Health Research University of Birmingham Birmingham United Kingdom; ^2^ Community Medicine Department Faculty of Medicine Suez Canal University Ismailia Egypt

**Keywords:** Internet, information-seeking behavior, computer literacy, surveys and questionnaires, Egypt

## Abstract

**Background:**

Over the last decade, the Internet has become an important source of health-related information for a wide range of users worldwide. Yet, little is known about the personal characteristics of Egyptian Internet users who search for online health information (OHI).

**Objective:**

The aim of the study was to identify the personal characteristics of Egyptian OHI seekers and to determine any associations between their personal characteristics and their health information-seeking behavior.

**Methods:**

This cross-sectional questionnaire study was conducted from June to October 2015. A Web-based questionnaire was sent to Egyptian users aged 18 years and older (N=1400) of a popular Arabic-language health information website. The questionnaire included (1) demographic characteristics; (2) self-reported general health status; and (3) OHI-seeking behavior that included frequency of use, different topics sought, and self-reported impact of obtained OHI on health behaviors. Data were analyzed using descriptive statistics and multiple regression analysis.

**Results:**

A total of 490 participants completed the electronic questionnaire with a response rate equivalent to 35.0% (490/1400). Regarding personal characteristics, 57.1% (280/490) of participants were females, 63.4% (311/490) had a university level qualification, and 37.1% (182/490) had a chronic health problem. The most commonly sought OHI by the participants was nutrition-related. Results of the multiple regression analysis showed that 31.0% of the variance in frequency of seeking OHI among Egyptian adults can be predicted by personal characteristics. Participants who sought OHI more frequently were likely to be female, of younger age, had higher education levels, and good self-reported general health.

**Conclusions:**

Our results provide insights into personal characteristics and OHI-seeking behaviors of Egyptian OHI users. This will contribute to better recognize their needs, highlight ways to increase the availability of appropriate OHI, and may lead to the provision of tools allowing Egyptian OHI users to navigate to the highest-quality health information.

## Introduction

The use of the Internet as a source of health information has increased considerably over the last decade worldwide [1]. Many Internet users have switched to the Internet to obtain information about health and health care [2,3]. The Internet World Stats report indicates that the number of Internet users in Egypt has increased from 29.8 million in June 2015 (32.6% of the population) to 33.3 million in June 2016 (37.0% of the population) [4]. Most of this growth in Internet use has been around social activities (social media and email), educational activities, and playing games. However, the number of Egyptian Internet users who search for health-related information has increased from 29.7% in 2012 to 32.4% in 2013 from the total Egyptian Internet users as reported by the Egyptian Ministry of Communication and Information Technology (MCIT) [5]. Whereas most health-related websites funded by the Egyptian Government are targeting professional users, there is now a range of private Arabic language websites providing general health information to users; however, their quality tends to be variable [6].

Online health information (OHI) seekers are defined as “Internet users who search online for information on health topics, whether they are acting as consumers, caregivers or patients aiming to take more control of their health” [7]. The variability of personal characteristics such as age, gender, race or ethnicity, and income levels can be a predictor for using the Internet as a source of health information [8-10].

Using the Internet to search for health information has many advantages: easy access, availability of a wide range of illustrated and audiovisual health resources, as well as providing an opportunity to ask experts’ opinion [11]. Furthermore, its anonymity enables users to express their health problems and share experiences privately with other people in a similar situation [12]. The Internet can contribute to the improvement of social interaction, better coping with life situations, more knowledge about particular diseases, emotional relief, and improvement in clinical outcomes for health information seekers [13]. Despite the advantages of using OHI, the Internet may also present a great risk for consumers when they are not aware of the quality standards of the obtained health information [14,15]. Studies have reported that health information available on the Internet is sometimes scientifically incomplete or inadequate [16,17]. When the health information obtained from the Internet is of questionable quality, it may negatively influence the doctor-patient relationship [18,19]. The wide variability of resources on the Internet and the commercial interest of some providers can cause confusion to the user and make it difficult to locate accurate and reliable health information [17]. Additionally, there are some barriers to access the Internet such as lack of computer skills, lower levels of education, and geographic location [20,21].

Public awareness of the usefulness of OHI has risen in low- and middle-income countries as well as in the Arab world [22,23]. Although the number of Egyptian OHI seekers has increased, knowledge of personal characteristics of OHI seekers and their motives behind searching for OHI is still limited [5]. This study aimed to identify the association between personal characteristics of Egyptian OHI seekers and their health information-seeking behavior. This may help to improve ways of promoting efficient and appropriate OHI for users and harnessing the benefits of the Internet as a source of health information.

## Methods

Data for this cross-sectional questionnaire study was collected from June to October 2015. An electronic questionnaire was sent to registered users meeting the inclusion criteria (currently living in Egypt and aged 18 years or older; N=1400) of a widely used Arabic language health information website (6abibak.com) by the website administrator. Two reminders were sent to the selected participants in order to improve the response rate. The invitation included information about the aim of the study and reassurance about the anonymity of collected data. After completing the consent form, participants progressed to complete the Web-based questionnaire. The questionnaire was developed based on previous studies[16,17,24,25]. The questionnaire was divided into the following three sections:

1. Sociodemographic characteristics: this included age, gender, levels of education, employment status (employed or not employed), marital status, and whether participants were covered by a health insurance scheme or not
2. Health status: participants were asked whether they had any chronic health problems and to rate their self-reported general health status on a scale ranging from poor to excellent
3. Health information-seeking behavior: participants were asked about their main source of health information, the frequency of using OHI (hours/ week), rating the quality of the OHI obtained, health topics they had searched for on the Internet, and whether they had made any behavioral change resulting from the obtained OHI.

The study was approved by the Ethics and Research Committee of The Faculty of Medicine, Suez Canal University, Egypt.

## Statistical Analysis

Statistical analysis was performed using SPSS statistical software (version 22; SPSS Inc). Descriptive statistics (eg, means and standard deviations) were used in the initial data analysis. Chi-square test was used to identify associations between different personal characteristics of the study participants and the use of Internet as the main source of health information. The level of statistical significance was set at *P* value<.05.

Standard multiple regression analysis was carried out to assess the contribution of personal characteristics (the independent variables) to the frequency of using OHI among the participants as a dependent variable. Independent variables were presented as a sequence of nested models. Model 1 included age, gender, and educational level. In model 2, we added self-reported general health and having a chronic health problem. In model 3, we added using the Internet as the main source of health information. In model 4 we added any self-reported behavioral changes due to the obtained OHI.

**Table 1:** Sociodemographic and health-related characteristics of study participants (N=490).

Sociodemographic characteristics		n (%)
**Age (years)**		
	18-20	36 (7.3)
	20-35	242 (49.4)
	35-50	181 (36.9)
	≥50	31 (6.4)
**Gender**		
	Male	210 (42.9)
	Female	280 (57.1)
**Highest level of education**		
	Primary education	69 (14.2)
	Secondary education	110 (22.4)
	University degree (undergraduate or postgraduate)	311(63.4)
**Social status**		
	Never married before	187 (38.2)
	Married	251 (51.2)
	Divorced or widow or widower	52 (10.6)
**Employment**		
	Employed	302 (61.6)
	Unemployed^a^	96 (19.6)
	Student	92 (18.8)
**Self-reported general health**		
	Excellent	95 (19.3)
	Good	282 (57.6)
	Poor	113 (23.1)
Having a chronic health problem	Yes^b^	182 (37.1)
Having health insurance cover	Yes	330 (67.3)

^a^Unemployed also included housewife or retired.

^b^Having one or more chronic health problems.

## Results

A total of 490 participants completed the Web-based questionnaire with a response rate of 35.00% (490/1400).

### Sociodemographic Characteristics

The main sociodemographic characteristics of study participants are summarized in Table 1. The mean age of the participants was 36.8 years. The number of female participants (57.1%, 280/490) was marginally higher than the male participants (42.9%, 210/490); 63.4% (311/490) were university graduates, and 61.6% (302/490) were employed.

### Self-Reported Health Status

About 76.9% (377/490) participants rated their general health as good or excellent; meanwhile, only 23.1% (113/490) rated it as poor. Of all the participants, 67.3% (330/490) reported that they had health insurance coverage (either private or government-based coverage). Additionally, 37.1% (182/490) of the participants reported one or more chronic health problems.

### Health Information-Seeking Behavior

The mean time spent searching for OHI was 1.58 (SD 2.13) hours/ week (Table 2). Of all the respondents, more than half (55.4%, 271/490) considered the Internet as a main source for health information, followed by consulting physicians (30.7%, 151/490), whereas TV and radio were the least commonly used sources for health information (3.9%, 19/490). The main reasons for preferring OHI were easy access (47.2%, 231/490) and getting more information about specific health problems (30.8%, 151/490),whereas only 11.6% (57/490) participants preferred OHI because of its ability to preserve the user’s anonymity.

**Table 2:** Distribution of the online health information (OHI)-seeking behavior of study participants (N=490).

OHI^a^-seeking behavior of the participants		n (%)
**Main source of health-related information^b^**		
	Internet	271 (55.4)
	Physicians	151 (30.7)
	Family members or friends or colleagues	49 (10.0)
	Radio or TV	19 (3.9)
	Others	79 (16.1)
**Average time spent on health information Web pages (hour/week)**		
	<2	226 (46.1)
	2-5	150 (30.7)
	>5	114 (23.2)
**Main reason behind preferring OHI**		
	Easy access	231(47.2)
	Improving understanding of a specific health problem	151 (30.8)
	Anonymity	57 (11.6)
	Recommended by family, friend, or physician	51(10.4)
**Web pages visited for health-related information**		
	Scientific societies	111 (22.6)
	Websites recommended by search engines such as Google, Yahoo, etc.	163 (33.3)
	Websites related to Ministry of Health or universities	81 (16.5)
	No special preference	135 (27.6)
**Participant rating of the OHI quality**		
	Excellent	172 (35.2)
	Good	235 (47.9)
	Fair	83 (16.9)
	Poor	0
**Looking for OHI**		
	For themselves	262 (53.5)
	For someone else	150 (30.6)
	Both	78 (15.9)
**Search topics**		
	New health issue	126 (25.7)
	Longstanding health issue	152 (31.0)
	Both	212 (43.3)
**Preferred search location for OHI**		
	Home	309 (63.1)
	Work	94 (19. 3)
	Others^c^	87 (17.6)

^a^OHI: online health information.

^b^Multiple responses allowed.

^c^Others include public places and Internet cafe.

**Table 3:** Cross-tabulations between personal characteristics of the study participants and using the Internet as a main source of health information(N=490).

Personal characteristics		n	Using the Internet as a main source of health information n (%)	*P* value^a^
**Age (years)**				
	<35	278	208 (74.8)	.001
	≥35	212	129 (60.8)	
**Gender**				
	Male	210	103 (49.1)	.01
	Female	280	168 (60.0)	
Having chronic diseases^b^	Yes	182	121 (66.5)	.001
Reporting behavioral changes after obtaining OHI^c^	Yes	283	173 (61.1)	.002

^a^Result is significant at  *P*<.05.

^b^Having one or more chronic health problems.

^c^OHI: online health information.

**Figure 1:**
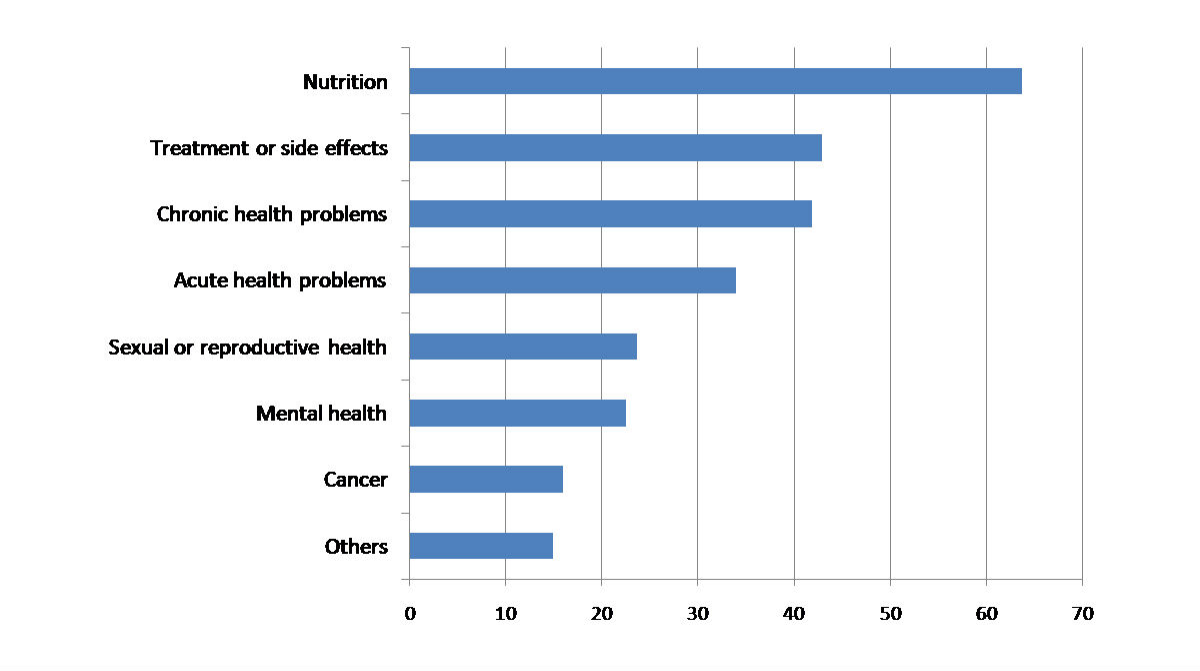
Common health topics sought on the Internet by the study participants (%).

The majority of participants (83.1%, 407/490) rated the quality of OHI as good or excellent, and 16.9% (83/490) rated it as fair, whereas none rated it as poor. About 53.5% (262/490) of participants reported looking for health information for their own health issues, whereas 30.6% (150/490) were looking for health information for someone else, for example, family member or a friend (Table 2).

Table 3 compares personal characteristics of the participants and their use of the Internet as the main source of information, which was significantly higher among female participants (χ^2^_1_=5.8, *P*=.01), younger age group participants (χ^2^_1_=10.4, *P*=.001), and participants who reported having chronic health conditions (χ^2^_1_=14.6, *P*=.001; Table 3). Using the Internet as the main source of health information was significantly higher among participants who reported behavioral changes from the obtained (χ^2^_1_=9.1, *P*=.002, Table 3).

Participants reported seeking OHI for one or more of a broad range of health topics. About 63.8% (312/490) of the participants indicated that they sought information about nutrition and 41.9% (205/490) of the participants reported using the Internet to find information about chronic health conditions, whereas 23.7% (116/490) searched for sexual health information and 22.6% (110/490) participants searched for mental health issues (Figure 1).

**Table 4:** Multiple regression analysis with frequency of using online health information (OHI) as dependent variable and personal characteristics of Internet users as independent variables (*P*<.05. statistically significant).

Personal characteristics	Model 1		Model 2		Model 3		Model 4	
	Beta^a^	SE^b^	Beta	SE	Beta	SE	Beta	SE
Age	−0.19	0.04	−0.19	0.04	−0.16	0.03	−0.16	0.03
Female (vs male)	.22	0.04	.21	0.04	.22	0.03	.20	0.04
Primary educated (vs college graduate)	.62	0.08	.09	0.06	.09	0.10	.09	0.08
Secondary educated (vs college graduates)	.12	0.09	.11	0.08	−0.12	0.09	.13	0.09
Poor general health (vs good)			.07	0.12	.07	0.10	.07	0.12
Fair general health (vs good)			−0.01	0.26	−0.05	0.26	.02	0.26
With chronic disease (vs without).			.06	0.02	−0.02	0.02	.03	0.08
Health insurance (vs noninsured)			.07	0.18	.06	0.18	.07	0.18
Internet as the main source of health information (vs not)					.12	0.19	.07	0.23
Behavior changes (vs no changes)							. 95	0.19
Constant	-	3.13	-	5.71	-	5.80	-	5.49
*F* value	18.02		12.80		12.71		11.94	
R^2^	0.23		0.28		0.29		0.31	

^a^Beta: unstandardized.

^b^SE: standard error.

Table 4 shows the results of the multiple regression analysis with personal characteristics as independent variables and the frequency of using OHI as a dependent variable. In model 1, the regression coefficient of the independent variables age (beta=−0.16), gender (beta=.02), and level of education (beta=.13) were statistically significant. The model showed that younger participants spent more time on searching OHI than older participants. Model 1 can predict 23.2% of the variance in using OHI among the participants **.**

Adding the self-reported general health status in regression model 2, a statistically significant increase in R^2^(from 0.23 to 0.28, *P*<.05) occurred. The direction and the significance effect of age, gender, and education remain the same as in model 1. Adding the use of the Internet as a main source of health information in regression model 3, a further statistically significant increase occurred in the variance in frequency of using OHI (R^2^=.29, *P*<.05). The regression coefficients were statistically significant for the following independent variables (*P*<.05): age, gender, levels of education, and having a chronic health problem.

Overall, 31.0% of the variance in frequency of using OHI can be predicted by the personal characteristics of Internet users (*F*=9.94, *P*<.05, Model 4). The multiple regression analysis showed that the main predictors are being female, of younger age, having a higher level of education, reporting good general health, having a chronic health problem, and using the Internet as a main source of health information.

## Discussion

### Principal Findings

In our study, we explored characteristics and behaviors of Egyptian OHI seekers. Our findings showed that the main personal characteristics associated with more frequent OHI searching were younger age, female gender, higher education, and reporting good general health.

The study participants represent a small sample of Egyptian Internet users who seek OHI. We have no data of those who declined to take part in the study or those who had not searched OHI at any given time. Accordingly, the results of the study can be generalized only to Egyptian OHI seekers. Participants’ answers to the questionnaire were self-reported and could not be independently verified. None of the participants rated the quality of OHI as poor, which may be because participants who took part in the study were active users of a health website.

In relation to the association between gender and OHI-seeking behavior, our findings show that female participants (57.1%) have looked for OHI more frequently than male participants (42.9%). This may arise from curiosity about their own health, as women seem to be more involved in decisions about their health [26]. Similar results were reported by Rice et al, which shows that the female gender is an important predictor for OHI-seeking behavior [12]. However, this should be seen in the context that according to a 2013 report from the Egyptian MCIT, a minority (44.4%) of Egyptian Internet users were females, whereas 56.6% were males [5].

The report also stated that 72% of Egyptian Internet users ranged between 15-44 years old, whereas only 19% were aged 45 years or older [5]. In this study, we found that younger participants sought OHI more frequently than older participants. The reason for that may be that younger OHI seekers are more familiar with Internet use [25]. Similarly, Nolke et al reported that the probability of seeking OHI in German participants decreased with increasing age [27].

Participants’ level of education also had a considerable impact on OHI seeking behavior. In our study, participants with higher educational level spent more time seeking for OHI. This is to be expected given that 73% of Egyptian Internet users were university graduates [5]. Cotten et al found that OHI seekers tended to have higher levels of education than those who did not seek OHI, which confirms a link between educational level and OHI seeking [28].

Our findings also show that OHI seekers with good self-reported general health were likely to search for OHI more frequently than those with poor general health. Maybe because more than 50% of the participants were less than 35 years old, this meant that they were more likely to be in good general health. Similarly, Weaver et al found that healthier people use OHI more frequently in a proactive manner for health promotion or to maintain a healthy lifestyle [8]. However, Cotten et al found that healthier persons seek OHI less frequently than those with poor general health [28].

We found that people who reported one or more chronic health problems were more likely to search for OHI more frequently than people without any chronic health conditions. This link between having a chronic health problem and the frequency of OHI seeking is supported by some researchers, whereas others did not find a significant association [10,29-32]. The reason for this discrepancy is not clear but it might be argued that the two groups used OHI differently: healthy participants focused on improving their lifestyles, whereas people with chronic illness sought to improve their current health condition.

In this study, participants reported seeking OHI for a broad range of health topics including nutrition, sexual health, chronic health problems, and mental health. We found that nutrition-related health information is the most common topic sought on the Internet especially by female participants. This finding can be seen in the context of increased prevalence of nutrition-related health problems among Egyptian adults [33]. As reported in the DHS report in 2015, 30% of Egyptian adults are overweight or obese, and 6.8% of Egyptian children under the age of 5 years were malnourished (eg, micronutrients deficiency or anemic) [34,35].

Despite the ease of finding health information on the Internet, it may be difficult to judge the reliability of this health information. This may explain the variability in the level of trust in OHI [17]. Most OHI seekers in Arabic countries rely on health sites maintained mainly by the private sector rather than by ministries of health or other public services. Arabic language health information websites need a substantial improvement of their quality in order to improve their trustworthiness [6]. However, most of our participants rated the quality of the OHI accessed as “good” or “excellent.” For some, this may be linked to lack of trust in health professionals as much as increased trust in OHI sites.

### Conclusions

The Internet has become an important tool with the potential to improve information dissemination and health care delivery to consumers. Continuing efforts to maximize the potential of this tool could have great value for users. The great range of health topics as well as the easy access to OHI has made the Internet a major and growing source of health information for Egyptian Internet users. We found that personal characteristics of OHI seekers such as younger age, females, higher levels of education, and having generally good health make a difference in how frequently they access the Internet for health information. An understanding of personal characteristics may inform education of OHI seekers to help them to take an active role in improving their health and engaging with treatment plans; however, more research is needed to identify whether these characteristics can be confirmed in a representative sample of Egyptian Internet users.

It is also concerning that most providers of Arabic language OHI are privately owned and therefore, their priority may be increasing advertising revenue rather than providing reliable information. There is a need for public funding for quality controlled websites, for example, equivalent to the United Kingdom’s National Health Service (NHS) Choices. Cooperation from health care providers is also needed in supporting their patients with reliable resources and assisting them to properly evaluate the quality of information available on the Internet in order to promote efficient, reliable, and appropriate OHI use for Internet users.

## Abbreviations

OHI: online health information
MCIT: Ministry of Communication and Information Technology
NHS: National Health Service
